# A Standard-Based Internet of Things Platform and Data Flow Modeling for Smart Environmental Monitoring

**DOI:** 10.3390/s21124228

**Published:** 2021-06-20

**Authors:** Tércio Filho, Luiz Fernando, Marcos Rabelo, Sérgio Silva, Carlos Santos, Maria Ribeiro, Ian A. Grout, Waldir Moreira, Antonio Oliveira-Jr

**Affiliations:** 1 Institute of Biotechnology (IBiotec), Federal University of Catalão (UFCAT), Catalão 75705-220, Brazil; sergio@ufg.br; 2 Graduate Program in Science and Technology (PPGCET), Federal University of Catalão (UFCAT), Catalão 75705-220, Brazil; luizfernandoelias@gmail.com (L.F.); marcosrabelo@ufg.br (M.R.); 3 Campus Palmas, Federal Institute of Education, Science and Technology of Tocantins (IFTO), Palmas 77021-090, Brazil; carlosedu@ifto.edu.br; 4 Institute for Systems and Computer Engineering, Technology and Science (INESC-TEC), 4200-465 Porto, Portugal; maria.r.ribeiro@inesctec.pt; 5 Department of Electronic and Computer Engineering, Faculty of Science and Engineering, University of Limerick, V94 T9PX Limerick, Ireland; Ian.Grout@ul.ie; 6 Fraunhofer Portugal AICOS, 4200-135 Porto, Portugal; waldir.junior@fraunhofer.pt (W.M.); antoniojr@ufg.br (A.O.-J.); 7 Institute of Informatics (INF), Federal University of Goiás (UFG), Goiânia 74690-900, Brazil

**Keywords:** IoT platform, IEEE 1451 standard, smart environmental monitoring, data flow modeling

## Abstract

The environment consists of the interaction between the physical, biotic, and anthropic means. As this interaction is dynamic, environmental characteristics tend to change naturally over time, requiring continuous monitoring. In this scenario, the internet of things (IoT), together with traditional sensor networks, allows for the monitoring of various environmental aspects such as air, water, atmospheric, and soil conditions, and sending data to different users and remote applications. This paper proposes a Standard-based Internet of Things Platform and Data Flow Modeling for Smart Environmental Monitoring. The platform consists of an IoT network based on the IEEE 1451 standard which has the network capable application processor (NCAP) node (coordinator) and multiple wireless transducers interface module (WTIM) nodes. A WTIM node consists of one or more transducers, a data transfer interface and a processing unit. Thus, with the developed network, it is possible to collect environmental data at different points within a city landscape, to perform analysis of the communication distance between the WTIM nodes, and monitor the number of bytes transferred according to each network node. In addition, a dynamic model of data flow is proposed where the performance of the NCAP and WTIM nodes are described through state variables, relating directly to the information exchange dynamics between the communicating nodes in the mesh network. The modeling results showed stability in the network. Such stability means that the network has capacity of preserve its flow of information, for a long period of time, without loss frames or packets due to congestion.

## 1. Introduction

The use of technology for environmental monitoring has become an important tool for the management of public health and the inspection of flora. In addition, such a tool allows studies focusing on climate and micro-climate analyses, and weather forecasting. These studies can provide essential knowledge for a better management of resources and planning in a specific region. Possible areas that can benefit of such knowledge include agriculture, livestock, fish farming, apiculture, among others [1].

Atmospheric processes and factors have been studied by meteorologists and climatologists, respectively, with the focus on weather forecasting and characterization of the climate in macro- and micro-regions [2,3]. The factors of the weather in a region, also called meteorological variables, such as humidity, temperature, precipitation, and wind direction that have been analyzed over decades allow characterization of the climate according to the seasons of the year. This knowledge can be used in the management of resources and for planning in activities such as civil construction and crop growth. This knowledge also becomes important for the analysis of air pollution, its dispersion, concentration and possible interactions of the environment with living beings present in a given place. However, it is difficult to monitor the weather of a specific region for a long period of time to conduct a climate analysis without fully automating the data acquisition process. To narrow this gap, wireless sensors networks have been developed.

A wireless sensor network (WSN) can be defined as a network of devices, capable of measuring aspects of the environment and propagate the information collected through the network. The data are transmitted through the network nodes, possibly via multiple jumps until a master node, using a wireless interfaces. The data collected by a sensor network can be heterogeneous and distributed. In addition, the data are collected for a long time and are stored on a server automatically, which can form large data repositories. In a WSN project, specific factors must be taken into account: fault tolerance, scalability, low cost, low power consumption, programmability, and security. In this case, security regarding aspects such as access control, message integrity, confidentiality and protection against reproduction [4,5].

For the integration of networked devices, the internet of things (IoT) emerged as a new paradigm making objects connected via the Internet. The concept becomes increasingly possible due to the miniaturization of devices and transducers, in addition to the increased data transmission rate over the network. Such features result in a significant increase in the number of objects connected in a network, making them indispensable in people’s lives. The most used technologies for the development of networks based on IoT are radio frequency identification (RFID), WSN, middleware, cloud computing, and IoT application software [6]. IoT-based systems are applied in several areas: smart homes, smart cities, health care, energy management, environmental monitoring, and agriculture. Each application can use a specific protocol for IoT systems, such as hypertext transfer protocol (HTTP), transmission control protocol/user datagram protocol (TCP/UDP), extensible messaging and presence protocol (XMPP), message queuing telemetry transport (MQTT), and message queuing telemetry transport-sensor network (MQTT-SN). A standard that has been adapted to the IoT is the IEEE 1451 standard.

**Figure 1:**
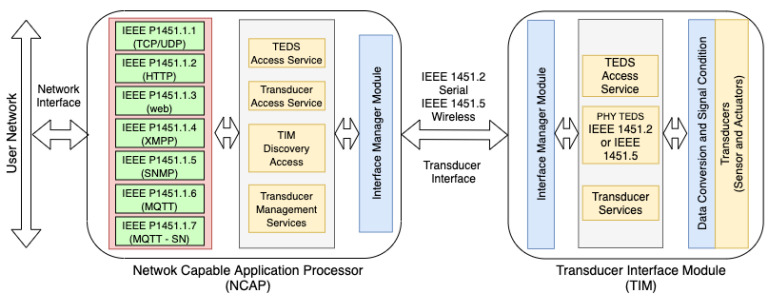
Network topology developed.

The IEEE 1451 standard provides functionalities, protocols, communication interfaces, and plug-and-play features through transducers electronic data sheet (TEDS). Basically, the IEEE 1451 standard specifies three entities: network capable application processor (NCAP), transducer interface module (TIM), and Client. NCAP is a network gateway that receives data from the external network, processes the data, and forwards the processed data to the internal network based on one of the subcommittees of the IEEE 1451 standard. TIM, is a network module that contains a communication interface with NCAP, sensors/actuators, processing unit, signal conditioning circuit and TEDS. TEDS are the TIM module information stored in a non-volatile memory. The client is the network user or an end device. With the advent of transducer networks, the client entity is defined according to: IEEE 1451.1.1 (TCP/UDP), IEEE 1451.1.2 (HTTP), IEEE 1451.1.3 (web interface), IEEE 1451.1.4 (XML extensible markup language (XMPP)), IEEE 1451.1.5 (simple network management protocol (SMNP)), IEEE 1451.1.6 (MQTT), and IEEE 1451.1.7 (MQTT-SN) [7]. In this context, it is possible to apply the IEEE 1451 standard and the IoT concept to smart transducer networks, increasing the flexibility of devices to access data, performance, insertion of new devices accessing data and decreasing costs [8]. Figure 1 shows the model define by IEEE 1451 for development of transducers network.

This paper proposes a standard-based IoT platform and data flow modeling for smart environmental monitoring employing ZigBee interfaces with the MQTT protocol (IEEE 1451.1.6). Thus, an NCAP/Broker was developed for the integration of the two network models and the collection of data from the WTIM modules over the Internet. The NCAP network node converts the data to the Internet using the publish/subscribe standard. The network was applied in the field for the tests of communication and reading of the data at two weather stations developed with ZigBee transmitters. Thus, it was possible to carry out the network analysis in practice through data collection using the MQTT protocol and the IEEE 1451 standard according to Figure 1.

To validate the proposed network model, it is possible to use theoretical/computational models that analyze the degree of parity between the theoretical model and the experimental model. One of the central issues is the control of network congestion and the study of techniques that are able to prevent its emergence. In general, the control mechanisms take into account techniques that manipulate the data flow between the nodes of the [9] network. One way to model the flow is to consider the network as a dynamic system [10]. Among the various topics that can be addressed, the control of flow congestion has aroused interest. This is due to the fact that congestion control strategies are built through dynamic models whose complexity is not yet fully understood [10,11,12]. Thus, concepts such as phase portrait, stability, fixed point, and bifurcations, widely used in classical dynamic systems, can be applied to analyze the behavior of the data flow in the network [12]. In addition to the development of the NCAP/Broker network node, the contributions of this work are as follows:

- development of a mesh sensor network based on the IEEE 1451 standard for IoT;
- data flow analysis of the internal network;
- analysis of network communication based on the RSSI signal;
- analysis of packet loss between communication networks; and
- modeling the network data flow for congestion analysis.

The remainder of this paper is organized as follows. Section 2 discusses related works and highlights the contributions presented in this research. Section 3 shows the network developed, the NCAP, wireless transducer interface module (WTIM), and commands. Section 4 details the wireless sensor network implemented in the field, and Section 5 presents the network data flow modeling for analysis. The last section, Section 6, provides conclusions to the paper.

## 2. Related Work

The application of IoT networks [13] is increasingly becoming part of the people lives, carrying out the control of complex systems [14], environmental monitoring [15], precision agriculture [16,17], digital farming [18], digital health [19], and smart homes. Several researches have employed IoT networks to collect big data for analysis and complex decision making. In [20], the authors use a multidimensional spatial scan technique to discover household movement patterns, in order to help in the development and planning of public policies. The research in [21] reviews studies of IoT applied to smart cities and sustainable cities, including research aimed at conceptual, analytical, and overarching levels, as well as research on specific technologies and applications. In [22], the big data of of supply chains in manufacturing industries and artificial neural networks are used to identify predictors of sustainable business in emerging economies. In [23], a social-ecological model is developed to understand how ecosystems services drive urban growth. The research in [24] links IoT to Industry 4.0, deriving an empirical formulation to establish a mapping between Industry 4.0 attributes and sustainability goals. For a more detailed overview of smart environmental monitoring IoT-based solutions and platforms, we direct the reader to the work in [25].

In addition to the importance of IoT in providing high-value applications in various areas of knowledge, it is essential that the IoT infrastructure uses open standards in a way that is easily replicable, maintainable, and scalable. Currently, an open standard that complies with these characteristics is IEEE 1451. Several studies have been carried out using the IEEE 1451 standard and IoT systems, as, for example, in [26], where the authors presented a TIM using two communication interfaces. The highway addressable remote transducer (HART)-based module and TIM modules are based on the IEEE 1451 standard. The HART is a hybrid analog/digital industrial automation protocol. The HART-based module was coded using very high speed integrated circuit (VHSIC) hardware description language (VHDL) and implemented using the field programmable gate array (FPGA) to communicate with devices through the use of the universal asynchronous receiver/transmitter (UART) protocol.

In [8], the combination of IoT concepts and the active subcommittees of the IEEE 1451 standard are considered. They also presented an integration of the IEEE 1451 standard using IoT concepts and a new proposal for how the transducer electronic data sheet (TEDS) is referred to as the health electronic data sheet (HEDS). HEDS is an adaptation of TEDS for a health area, making it possible to distribute health assessment data at all levels of a system hierarchy.

The authors of [27] presented the IoT architecture in intelligent environments and a comparison between the technologies used for environmental monitoring. The authors presented technologies and sensors, such as the Arduino UNO, DHT11 sensor, and the ESP8266 wireless transmitter. The study carried out by the authors on intelligent monitoring using IoT facilitates the development of future work by other researchers.

In another work [28], the authors presented a new concept for services critical to IoT defined as critical IoT (CIoT). In more critical services, IoT protocols become vulnerable having to work with a communication prioritization scheme. Thus, a new criticality level (CL) and a security level (SL) are proposed to categorize and prioritize various types of relevant transmission. The authors also describe the use of the NCAP-IEEE 1451 standard, WTIM and TEDS for a mobile cloud computing (MCC) connection solution. In this way, it was possible to present a prioritization scheme involving the indices of CL and SL proposed to solve the problem related to latency tolerant.

In [14,29,30], the authors developed a wireless sensor network based on the Zigbee standard to different environments and performed analyses of performance. For example, the authors of [29] introduced an open-source wireless mesh network (WMN) module that integrated the functions of network discovery, automatic routing control, and transmission scheduling. In [14], a smart grid architecture-based system was developed that used a low-power Zigbee mesh network in response to adaptive traffic on the road. The sensor network was implemented and tested in a real environment. The system demonstrated that is capable of energy savings depending on the variations in daylight hours between summer and winter. In [30], the authors proposed a linear sensor network to deployment at oil and gas pipeline using a custom sensor board accompanied with algorithms to solve network creation, leak interrupt detection, and routing of high-priority messages with reliability while keeping network active. The authors consider the development of the network to be low energy consumption, latency, access to sensors/actuators, and data reliability.

Projects have been developed in the area of IoT using the IEEE 1451 standard, carrying out the monitoring of the sequence of painting and washing tubs [31]. In [32], TIMs were developed using an MSP430F5529 board and a Raspberry Pi as the NCAP. Another Raspberry Pi was used as an MQTT broker based on IEEE 1451.1.6. The author’s analysis was realized based on three transducers: temperature sensor, voltage sensor, and light-emitting diode (LED). The work also presents the description of TEDS and the common characteristics: identification, representation, communication, life cycle, function, and interoperability.

In [33], the development of a semantic sensor web (SSW) as a combination of sensor networks, web services, database, and semantic web technologies for monitoring environmental conditions is shown. The data were collected in the environment, stored in a database, and made available to the software through an application programming interface (API). In order to provide the data obtained by the API, a semantic web was used which created the data interoperability in a system such as resource description framework (RDF), a language used to process metadata, the SPARQL protocol, and RDF query language (SPARQL) which is an integration class between RDF and hypertext preprocessor (PHP).

Several models have been presented in the literature to deal with network congestion. In [10], results on flow stability are presented through the design of congestion controllers, using models of dynamic systems to describe the network. Due to the number of parameters involved in network models, it is common for bifurcations to appear at equilibrium points. In [34], Hopf bifurcation problems are studied in the context of congestion controller design.

In [10], a general multi-link/multi-source model for TCP/random early detection (RED) transfer protocols was proposed and it was demonstrated that the dynamic behavior of the packet flow is strongly influenced by the stability of the TCP/RED protocol. Ensuring a relationship between the stability of the system and the communication delay time between processes, allowing us to conclude that the increase in the delay time generates instability in the protocol.

In the aforementioned presented works, different types of wireless networks were identified and studies were carried out. However, they did not define a new description model for the external network based on the IEEE 1451 standard. Our work has used two models—the HTTP and MQTT protocols—according to Figure 1. In addition to the external network, the developed network presents the signal level between the nodes and the request and response time for each command, storing the data so that the network analysis can be performed. Our proposal also presents the formulation and analysis of the data flow of the developed network, differentiating it from the state of the art.

## 3. Our Proposal: IEEE 1451 Standard-Based IoT Platform

**Figure 2:**
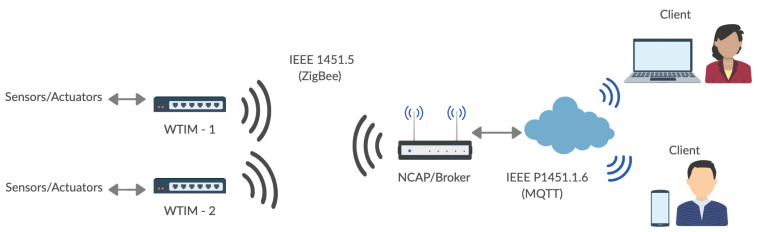
Network model developed.

The IEEE 1451 standard defines two network modules, called NCAP and WTIM, and a set of subcommittees for the definition of the communication interface, protocols, and TEDS [35,36]. The IEEE P1451.1.6 subcommittee specifies the characteristics of the NCAP and the communication with the external network using the MQTT communication protocol. The IEEE 1451.0-2007 standard describes common functions, communication protocols and TEDS, that is, descriptions of the transducers stored in a non-volatile memory in WTIM. The IEEE 1451.5-2007 standard specifies wireless network communication, whether WiFi, ZigBee, or Bluetooth. In this project, the ZigBee interface was used to communicate between NCAP and WTIM for internal network, and to external network MQTT-IEEE 1451.1.6. Figure 2 shows the network model developed for the collection of environmental data and analysis.

### 3.1. Network Capable Application Processor—NCAP (IEEE 1451.1.6)

The NCAP is a node with local processing capacity, capable of receiving data from an external network and converting the data to the internal network based on the IEEE 1451 standard, thus performing the reading/control of the transducers connected to TIM. For this, the NCAP node has the characteristic of identifying the type of network to which it is connected, thus giving the concept of interoperability, in addition to abstracting information of the type of connected transducer, facilitating the introduction of the plug-and-play operation mode [37].

NCAP was developed with the objective of creating an autonomous and expandable network that can carry out data collection for a long period of time. It has the functionality of discovering WTIM nodes in a plug-and-play way, reading sensors through WTIM nodes at predefined time intervals, storing data, and transmitting to an external network using the MQTT protocol.

The WTIM’s have a logical functionality in which the objective is to receive frames from the NCAP network node, verify that the frame corresponds to the WTIM and, if so, the frame is decoded and the command is carried out. If the command is not for the given WTIM, the frame is forwarded to the next WTIM. For the external network, the MQTT protocol was defined and the Mosquitto server was installed and configured on the NCAP network node. The NCAP protocol manager was developed in Python, making the requests for WTIM’s modules on the internal wireless ZigBee network and forwarding it to the MQTT broker. Thus, in turn, it makes a publication on the external network for forwarding to customers. Thus, the development of NCAP was divided into two levels: hardware and software. Section 3.1.1 presents a hardware and interface configuration, and Section 3.1.2 describes the software.

#### 3.1.1. Hardware and Interfaces

The NCAP was developed using the Raspberry Pi B+ made by the Raspberry Pi Foundation that has a Broadcom BCM2837 64bit ARMv8 Cortex-A53 Quad-Core 1.4 GHz, 1 GB of random access memory (RAM), and the following interfaces: Ethernet, Bluetooth, high-definition multimedia interface (HDMI) output, 4 universal serial bus (USB) ports, a graphics processing unit (GPU) and 40 pins, configured as 28 general purpose input/output (GPIO) pins (shared with serial peripheral interface (SPI), inter-integrated circuit (I2C). The transceiver module used was the XBee Pro S2C, manufactured by Digi International, having 2.4 GHz of frequency band, transmission rate of 250 Kb/s and can reaches a range up to 3200 m of communication distance according to the proprietary specification. The Raspberry and XBee Pro S2C communicate through connecting directly using the receive (Rx), transmission (Tx) from UART, and to turn on the X Bee Pro S2C, were used the pins 3.3 V and GND from the Raspberry Pi [38]. In this work, the security of the transmitted data is performed through the ZigBee standard using the XBee Pro S2C module. ZigBee security in the media access control (MAC) layer uses Encrypted Communication using Symmetric Keys, Frame Integrity through CRC Redundancy Checks and Frame Sequentiality. In addition to MAC layer security, ZigBee has Cryptographic Key Establishment, Key Transport, Frame Protection and Device Management. ZigBee uses 128-bit keys working with 3 types of keys, Network Key (used by all nodes in the network), Link Key (secret session keys, between connected devices) and Master Key (used to generate the key Link) [39].

The XBee Pro S2C modules were configured using the X-CTU software defined as coordinator and router. For the coordinator, the following parameters were changed: “PAN-ID” (definition of PAN ID); “CE”—Coordinator Enable (activated); “DL”—Destination Address Low (0 × FFFF) and “NI”—Node Indetification (Coordinator). For the router: “PAN-ID” (same as the coordinator); “CE”—Coordinator Enable (disabled); “DL” Destination Address Low (zero-(0)) and “NI”—Node-Indetification (WTIM). Thus, the commands are sent to the network nodes with the destination MAC, if the MAC is not from the node that received the command, it forwards to the next node. When the command has the same address as the network node, then is executed and returns with the response command to the coordinator using the coordinator node address or default address: “0 × 0000000000000000”.

#### 3.1.2. NCAP/MQTT Software

For the development of the logical part of the NCAP, the following tools were installed: Maria DB, Mosquitto, and Apache servers. The NCAP manager was developed in the Python language, using a digi.xbee.models package library to make the logical part of the communication between the Raspberry and the X Bee Pro S2C module. For sending data using the MQTT protocol, the paho.mqtt library was used.

**Figure 3:**
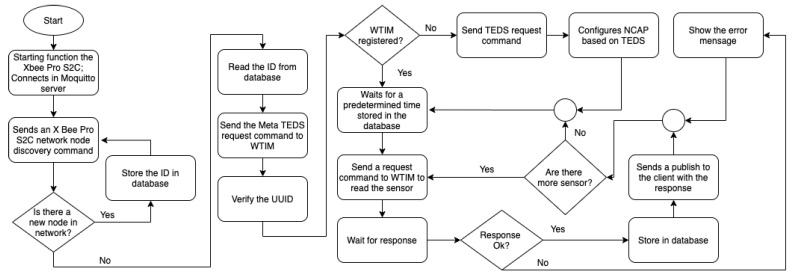
Flowchart of the logical part of the NCAP.

When the NCAP module is started, it sends a command on the network to add the WTIM modules. Upon receiving, each the WTIM modules returns the X Bee Pro S2C module ID, storing it in the database. New WTIM modules can be added at any time (plug and play) and, when the NCAP command occurs again, the new WTIMs will also have their IDs added to the database. If the ID is stored in the database, the NCAP sends a TEDS request command using the X Bee Pro S2C module ID; thus, it is possible to identify the WTIM module and perform the recognition of the transducers on the network. Figure 3 shows the flowchart of the NCAP network node and the recognition part of the WTIMs modules.

When the recognition of the WTIMs modules is complete, the NCAP automatically enters the data reading mode according to a predetermined time configured on the network coordinator. When the time is up, the NCAP sends a command to the first WTIM to request the data for each sensor. After that, the next module is verified in the database and forwards the request command. Upon receiving data from the internal network by the broker, the publication is made to customers. In the external network, the sender and the receiver are independent and can receive and send data simultaneously. The broker receives the data according to the predetermined time by the NCAP protocol manager and forwards it via MQTT using the publish according to Figure 2. An important characteristic of the broker is the filtering of messages, which is possible in 3 ways: topic, content, or type. In filtering by topic, the messages are sent in a hierarchical structure using the 8-bit unicode transformation format (UTF8) string in general. In content filtering, the messages are based on a filter language, and by type, messages are sent according to the type or class of the message. In this project, messages were sent using topics according to the following structure: Station _1/ Sensor/Temperature/Minimum, being divided into 4 levels, thus it was possible to define the sensors of each station. Figure 3 shows the flowchart of the NCAP protocol manager.

### 3.2. Wireless Transducer Interface Module—WTIM

**Figure 4:**
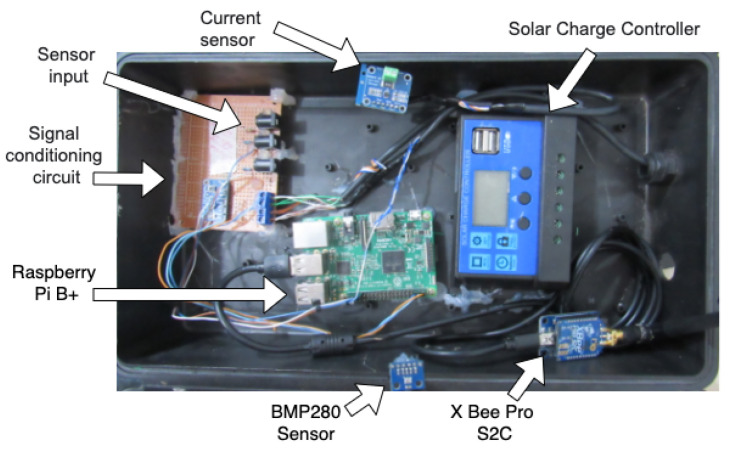
WTIM developed in the laboratory.

**Figure 5:**
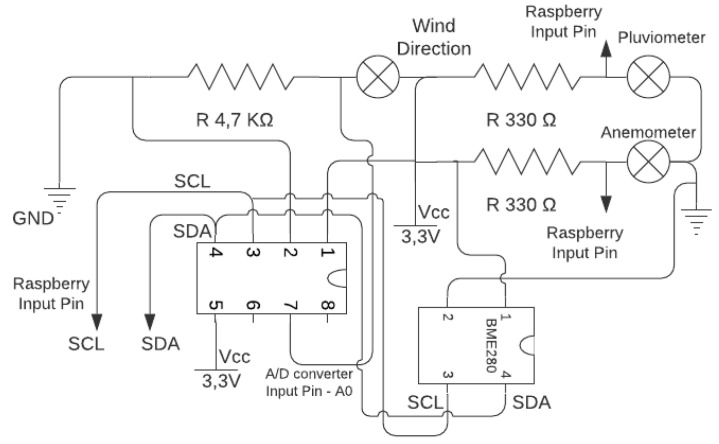
Signal conditioning circuit diagram.

The WTIM has characteristics similar to the NCAP, Raspbian operating system, and communication with the X Bee Pro S2C module is through the UART interface. However, the WTIM has sensors, signal conditioning circuits, and TEDS stored in files. Storage of TEDS files was specified by the name of each TEDS, such as Meta_TEDS.txt, TransducerChannel_TEDS.txt, UserTransducer_TEDS.txt, and Phy_TEDS.txt. The sensor module used was the BME280 that measures pressure, temperature and humidity. The characteristics of the BMP280 sensor are supply voltage of 1.8 V and 5V DC, I2C interface (up to 3.4 MHz), SPI (up to 10 MHz). In addition to the BMP280, the wind direction, wind speed, and rain sensor were used. Since the Raspberry Pi B + does not have an internal analog-to-digital converter (ADC), an external 16-bit ADC was used that communicates with the module using the I2C protocol. Figure 4 shows the WTIM node and Figure 5 presents the schematic of the developed electronic circuit.

**Figure 6:**
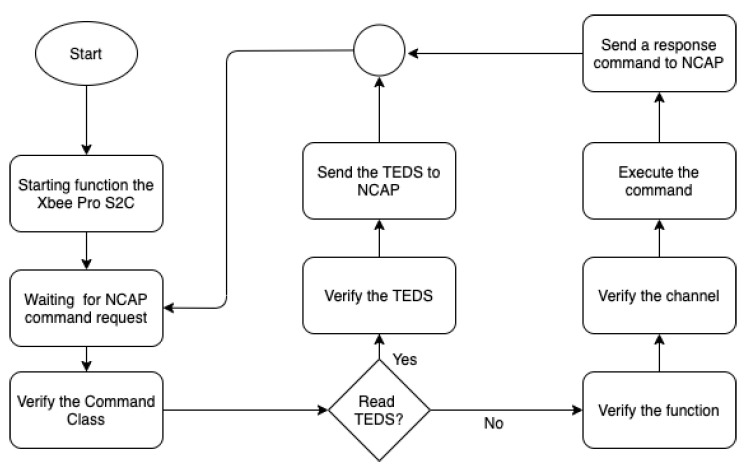
Flowchart of the logical part of WTIM.

The WTIM software is illustrated by the flowchart in Figure 6. The WTIM was developed using the Python language, using the same library for the development of NCAP, and communication with X Bee Pro S2C: digi.xbee.models package. When the WTIM is initialized, the UART interface, the interrupts and communications with sensor using the I2C are initialized. Then, the WTIM waits for an NCAP request. When it receives it, the frame is decapsulated and, then, is checked the command class based on the IEEE 1451 standard. If the class is 0 × 02, the command is for the manipulation of TEDS, if the class is 0 × 03, it checks the function of the command of access to transducers. The TEDS or sensor data is placed into the reply command based on IEEE 1451 standard, encapsulated in a ZigBee frame and devolved to the NCAP.

### 3.3. Request and Response Commands

The ZigBee and IEEE 1451.5 standards establish rules for communication between the devices envolved. In the ZigBee application layer, more specifically, in the application framework, the data transfer unit, called the API frame, is implemented.

The API frame is the basis of the communication between the devices of the ZigBee mesh network, once all devices communicate through frames. In the IEEE 1451.5 standard, communication between devices is done through IEEE 1451.5 frames. Thus, request and reply frames were implemented, referring to the sensor readings and the TEDS.

**Figure 7:**
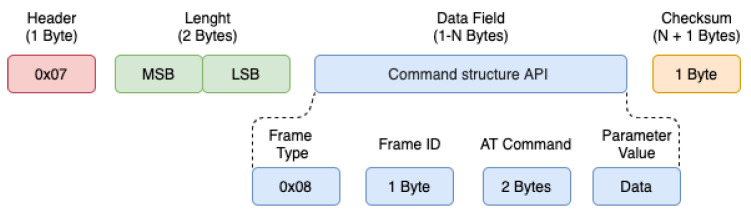
Generic protocol with AT command.

For the development of the mesh network using the ZigBee standard and IEEE 1451.5, the frames of both standards were joined. Thus, frames based on the IEEE 1451 standard are encapsulated in ZigBee frames and transmitted over the network. The API frame can contain several structures defined through the Type field. Each type of API frame contains a unique identifier, called the API Identifier ID (API). Through this identifier that the network and application layers recognize the characteristics of a frame sent or received. Two types of API frames were used to management and communication between the devices of the developed mesh network: AT command (0 × 08) and ZigBee transmission request (0 × 10). The “AT Command” frame was used to recognize the MAC ID of the X Bee Pro S2C connected to the Raspberry. The command is implemented on the main node of the network, that is, on the NCAP/Coordinator ZigBee node as shown in the Figure 7. The frame is used, generally, to configure or “read” parameters related to the XBee S2C module connected locally or remotely in the ZigBee mesh network. The generic structure of an “AT Command” frame is illustrated in Figure 7.

The frame request performs the exchange of messages between the devices of the ZigBee mesh network. The objective is to forward the IEEE 1451.5 frames through the data encapsulation regarding the acquisition and response in its data payload.

**Figure 8:**
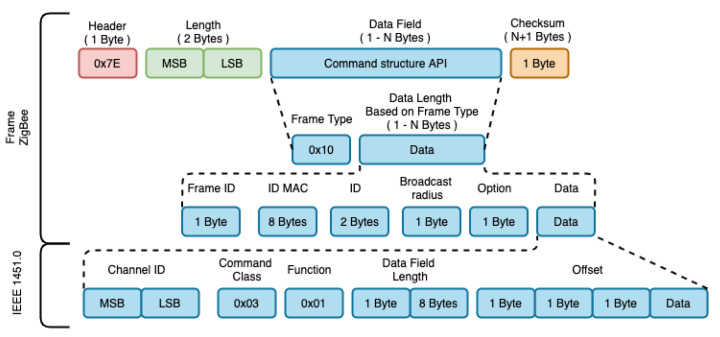
Request command.

In the transmission request frame shown in Figure 8, the byte that indicates the type of frame will be equal to 0 × 10 (request). Thus, according to type 0 × 10, bytes 6–13 are assigned to the MAC ID of the target device. Bytes 14–15 represent the short 16-bit address of the target device. Byte 16 corresponds to the broadcast radius of the message. Byte 17 is the transmission option field. Bytes 18–n corresponds to the data load of the frame. This is the field where the IEEE 1451.5 request frame will be stored. Figure 8 illustrates the encapsulation of a response frame. The difference in the structure of the response frame in relation to the allocation of a request frame is in the data payload field (18–n), where the IEEE 1451.5 response frame is stored. The IEEE 1451 command for accessing the transducers has the following structure: ID Channel (2 Bytes), Class (1 Byte), Function (1 Byte), Length (2 Bytes), and Data (N Bytes). The ID Channel defines the transducer channel; the Command Class specifies whether the command function is for access to the transducers, TEDS or, other configuration parameters; the Function specifies the task to be performed and, Length, the field size of data. Class 0 × 03 was used for access to transducers and 0 × 01 for access to TEDS. For Function, parameter 0 × 01 was used to read the transducers and 0 × 02 to read the TEDS. The TEDS tables are specified in the data field, being: 0 × 01-Meta TEDS, 0 × 03-Transducer TEDS, 0 × 0C-User TEDS and 0 × 0D-PHY TEDS.

**Figure 9:**
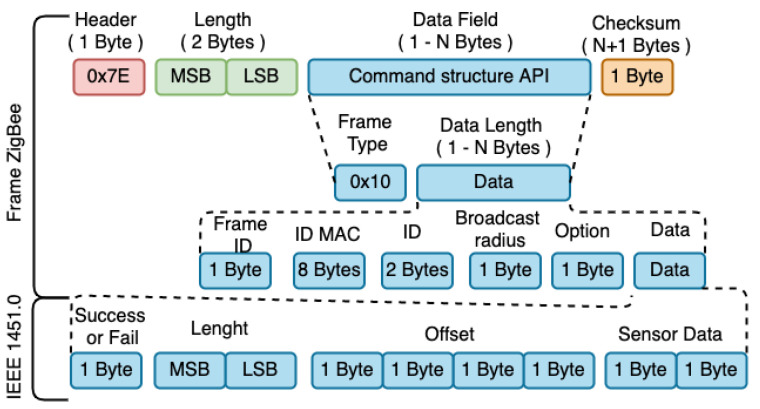
Response command.

The WTIM response is made by entering the coordinator’s MAC ID or the standard frame return value, being: “x00 × 00 × 00 × 00 × 00 × 00 × 00 × 00”. The response command defined by the IEEE 1451.0 standard is encapsulated in the ZigBee frame according to Figure 9 and forwarded to NCAP. The first Byte identifies whether the command was successful/failed, where different 0 × 00 means success. The next two Bytes, represents the size of the data field and the remaining Bytes, represents the data field with the command response.

To read the TEDS, the command follows the same model as the request and response for reading the sensors, inserting the IEEE 1451.0 command to read the TEDS in the data field of the ZigBee frame.

## 4. Mesh Network and System Description

**Figure 10:**
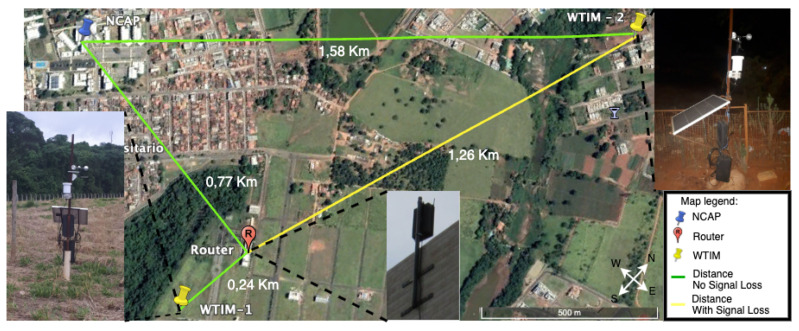
Network topology applied in the city.

The WTIMs nodes were distributed in different points of the city. Figure 10 shows the map with the distribution of this sensor network nodes and the distance between them. For WTIM_1, an intermediate router was inserted to communicate with NCAP due to the elevation of the soil and the woods. On WTIM_2, the communication was made directly with the NCAP coordinator, however, to improve the communication a 10 meter tower was buid and an X Bee S2C module installed at the top.

**Figure 11:**
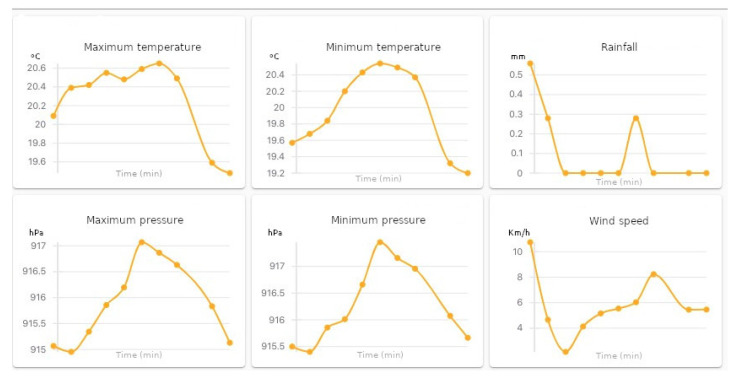
Reading of data via MQTT.

The coordinator makes the data available to the external network in two ways: using the HTTP protocol based on the IEEE 1451.1.2 standard and the MQTT, IEEE 1451.1.6 standard. The NCAP node sends reading requests once every 30 minutes through the ZigBee mesh network, receives the readings from WTIM nodes, and stores it in the database. Then, it sends to the client through the MQTT protocol. Thus, the data are visualized at the moment of reading through an MQTT client or through the web pages provided by the Apache server at the coordinator. Figure 11 shows the data received by coordinator to each sensor through to MQTT EXPLORER software.

The reading of the data can be achieved through a web page, in this case, the information is consulted in the database. In addition to the sensor data, received signal strength indication (RSSI) was collected before each request was sent to read the sensor and the time (Request/Response) to read data in the WTIM module. Table 1 shows the results obtained. To obtain the signal level, an AT command was used according to Figure 7. In the data field, the parameter “DB” was assigned.

## 5. Validation: Network Data Flow Modeling

This section presents an abstract modeling of the developed network. The justification that underlies this methodology lies in the fact that such models are efficient for measuring the flow of data between the components of the communication network. When approaching such a methodology, a central issue is the time delay that arises between us through the communication links. To deal with delay in the data flow, whether it is bit/s or packets/s, several strategies have been reported in the literature, among them, we mention control techniques, routing being one of the most used [9,40]. For these reasons, a better understanding of the nature and mechanisms that are responsible for the delay and how these mechanisms relate to the parameters of the network is important for a safe analysis of the stability of the network.

To proceed with the modeling, we consider the communication links as flow in networks where the bits are transmitted with a certain rate per second. This number is called the link’s transmission capacity. Generally, the link capacity is related to physical parameters of the communication channels, as well as to the interfaces related to it, that is, the data rate that the interface accepts to transmit. Thus, the management of the data flow in the network communication links has relevant effects on packet delay [9].

One of the established ways in the literature [9], to model data flow in the network is through transmission in a single row (multiplexing) with a first-come/first-served policy. In the language of queuing systems, customers arrive, with a random time, to be served by the processor. In the present work, NCAP is the service processor, while WTIMs are customers who arrive in line to be served. The probability distribution of time between successive arrivals and the probability of time in task processing are known.

**Figure 12:**
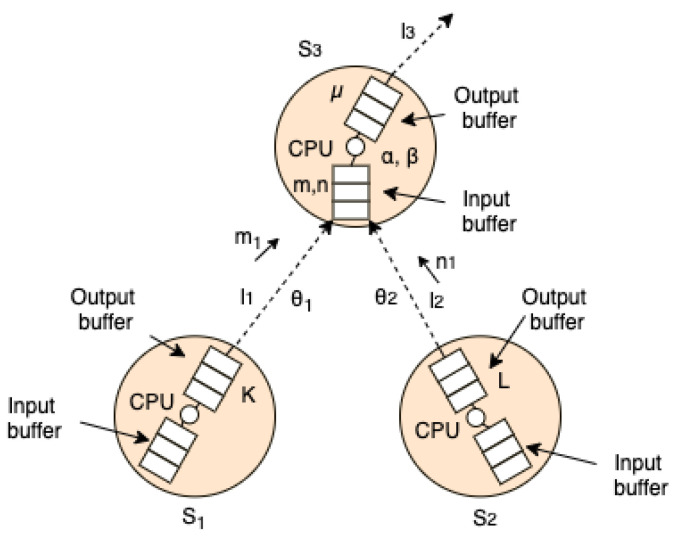
Architecture of the sensor networks.

In the context of the data network, clients represent the data packets transmitted through the communication links by WTIM’S while the server is represented by the data transmission carried out by NCAP. The service time corresponds to the packet transmission time made by NCAP is equal to $\frac{L}{C}$, where *L* is the length of the packet to be transmitted and *C* represents the communication capacity of the channel, presented in Figure 12.

The analysis of networks by queuing theory has the disadvantage of knowing in advance the probability distribution associated with the stochastic process that represents the dynamics of the random variables that describe the system. Another approach is to view the flow in the network as a dynamic system. In this direction, the network nodes can be seen as varying states in time that interact with each other through communication links [12,40]. The advantage of using the dynamic systems methodology is that all the concepts, tools and properties of the theory can be applied in the context of network flow. In this sense, a topic of extreme importance is the concept of stability that allows measuring the characteristic of the system in maintaining its operability, that is, maintaining the flow of communication between network nodes even with disturbances in the input signals.

In the literature, the contributions of dynamic systems in the analysis of stability and bifurcations have been shown to be quite intense. In what follows some contributions from recent research will be shown in order to contextualize the present work. Challenges in network research of underwater wireless sensors, reliability problems in transport flow in networks, routing protocols in networks, analysis of the dynamics of underwater sensor network congestion control models can be found at [12,41,42,43,44]. Themes involving stability analysis and bifurcation control can be found at [11,34,45,46,47]. The influence of the delay in network flow stability is found in [12,45,48]. Stability in impulse systems can be found at [49].

As previously mentioned, bifurcation stability issues are important issues to be considered in the data flow in the network, as branches of bifurcations can generate unstable equilibrium points and are responsible for the emergence of limit cycles and overflow, [50]. On the other hand, the presence of delay times can be a source of instability, [12,45,48,51]. To analyze the influence of the delay on the network stability, generally two approaches are considered, namely, the delay time can be known or not. Systems where the delay time is known are very rare to happen. Thus, to determine the delay, analytical methods are applied or sampling based on numerical algorithms based on models is performed. In the present work, the method of numerical algorithms will be applied, which will be done in the next subsection.

### 5.1. Model Description

In this section, we modeled the network, Figure 10, in an abstract way based on the Figure 12. In this model, WTIM’s and NCAP are represented, respectively, by $s_{i}$ and i=1,2,3; $m_{1},n_{1}$ represent, respectively, the density of information flow given in $bits/m^{3}$ or $packets/m^{3}$; the communication links with the transfer rate $r_{i},i=1,2$ represent the transfer rate given in bits/s or packets/s; K,L represent the communication capacity of the links $l_{i},i=1,2$; the bit loss rate due to proximity penalty between $s_{i},i=1,2,$ sensors are denoted by α,β, respectively; $\theta_{i},i=1,2$, represent the bit rate sent by the $s_{i}$ sensors, i=1,2; from the control point of view, the parameters m,n,μ are considered, representing respectively the loss of bits due to the stack overflow between nodes $s_{1}$ and $s_{3}$, the loss of bits between nodes $s_{2}$ and $s_{3}$ due to stack overflow, μ represents the bit loss rate due to stack overflow at node $s_{3}$. The control of network congestion is given by the following system of differential equations.

(1) $$\left\{\begin{array}{cc} {\dot{x}}_{1}\left(t\right) & =r_{1}x_{1}\left(1-\frac{x_{1}}{K}\right)-\alpha x_{1}x_{2}-mx_{1}x_{3}\\ {\dot{x}}_{2}\left(t\right) & =r_{2}x_{2}\left(1-\frac{x_{2}}{L}\right)-\beta x_{1}x_{2}-nx_{2}x_{3}\\ {\dot{x}}_{3}\left(t\right) & =\left(m_{1}x_{1}+n_{1}x_{2}\right)x_{3}-\mu x_{3}-\theta_{1}f\left(x_{1}\left(t-\tau \right)\right)x_{3}-\theta_{2}g\left(x_{2}\left(t-\tau \right)\right)x_{3}. \end{array}\right.$$

It is important to highlight that in the differential equation system, Equation (1), $\alpha ,\beta ,\mu ,m,n,\theta_{1}$ and $\theta_{2}$ together with the functions f,g are control parameters being, in the case of f,g used to manage the bit distribution of the $s_{1}$ and $s_{2}$ sensors.

### 5.2. Stability Analysis and Hopf Bifurcations

Important properties related to the solutions of Equation (1) can be obtained through the stability analysis of the linearized equations around the equilibrium points. Thus, proceeding to the linearization of Equation (1) we have

(2) $$\dot{x}=\mathrm{Ax}+Bx\left(t-\tau \right),$$

where τ represents the delay time in sending packages from both $s_{1}$ and $s_{2}$ to $s_{3}$; in this work, it is assumed that τ is common for both nodes $s_{i},i=1,2$; $A,B\in R^{3\times 3}$ are arrays given by

(3) $$\begin{array}{ccc} A & = & \left[\begin{array}{ccc} -r_{1}\frac{x_{1}^{*}}{K} & -\alpha x_{1}^{*} & -mx_{1}^{*}\\ -\beta x_{2}^{*} & -\frac{r_{2}x_{2}^{*}}{L} & -nx_{2}^{*}\\ m_{1}x_{3}^{*} & +n_{1}x_{3}^{*} & 0. \end{array}\right],\; \end{array}$$

(4) $$\begin{array}{ccc} B & = & \left[\begin{array}{ccc} 0 & 0 & 0\\ 0 & 0 & 0\\ -\theta_{1}f^{\prime }\left(x_{1}^{*}\right)x_{3}^{*} & -\theta_{2}g^{\prime }\left(x_{2}^{*}\right)x_{3}^{*} & 0 \end{array}\right], \end{array}$$

where the vector $x^{*}=\left(x_{1}^{*},x_{2}^{*},x_{3}^{*}\right)$ are the equilibrium points satisfying the following condition:

(5) $$\left\{\begin{array}{cc} \frac{r_{1}}{K}x_{1}+\alpha x_{2}+mx_{3}= & r_{1}\\ \left(\frac{nr_{1}}{K}-m\beta \right)x_{1}+\left(n\alpha -\frac{mr_{2}}{L}\right)x_{2}= & nr_{1}-mr_{2}\\ m_{1}x_{1}+n_{1}x_{2}-\theta_{1}f\left(x_{1}\right)-\theta_{2}g\left(x_{2}\right)= & \mu \end{array}\right.$$

Equation (5) can be rewritten as follows:

(6) $$\begin{array}{c} x_{2}=L\left(\gamma_{1}x_{1}+\gamma_{2}\right) \end{array}$$

(7) $$\begin{array}{c} m_{1}x_{1}+n_{1}L\left(\gamma_{1}x_{1}+\gamma_{2}\right)-\mu -\theta_{1}f\left(x_{1}\right)-\theta_{2}g\left(L\left(\gamma_{1}x_{1}+\gamma_{2}\right)\right)=0 \end{array}$$

(8) $$\begin{array}{c} x_{3}=\frac{r_{1}}{m}\left(1-\frac{x_{1}}{K}\right)-\frac{\alpha }{m}L\left(\gamma_{1}x_{1}+\gamma_{2}\right) \end{array}$$

where $\gamma_{1}$ and $\gamma_{2}$ are constants given by

$$\begin{array}{ccc} \gamma_{1} & = & \frac{\left(nr_{1}-Km\beta \right)}{K(mr_{2}-n\alpha L)}\\ \gamma_{2} & = & \frac{\left(mr_{2}-nr_{1}\right)}{\left(mr_{2}-n\alpha L\right)}. \end{array}$$

In Equations (6)–(8), just study the solutions to the Equation (7), as the equilibrium point $x^{*}=\left(x_{1}^{*},x_{2}^{*},x_{3}^{*}\right)$ is parameterized in $x_{1}.$ The characteristic of Equation (3) is given by

(9) $$\begin{array}{ccc} p(\lambda ,\tau ) & = & \lambda^{3}+b_{1}\lambda^{2}+b_{2}\lambda +b_{3}+\left(b_{4}\lambda +b_{5}\right)e^{-\lambda \tau } \end{array}$$

where

(10) $$\begin{array}{ccc} b_{1} & = & \frac{r_{1}x_{1}^{*}}{k}+\frac{r_{2}x_{2}^{*}}{l}\\ b_{2} & = & \frac{r_{1}r_{2}x_{1}^{*}x_{2}^{*}}{kl}-\alpha \beta x_{1}^{*}x_{2}^{*}+mm_{1}x_{1}^{*}x_{3}^{*}+nn_{1}x_{2}^{*}x_{3}^{*}\\ b_{3} & = & \frac{mm_{1}r_{2}x_{1}^{*}x_{2}^{*}x_{3}^{*}}{L}+\frac{nn_{1}r_{1}x_{1}^{*}x_{2}^{*}x_{3}^{*}}{K}-\alpha m_{1}nx_{1}^{*}x_{2}^{*}x_{3}^{*}-\beta mn_{1}x_{1}^{*}x_{2}^{*}x_{3}^{*}\;\\ b_{4} & = & -m\theta_{1}f^{\prime }\left(x_{1}^{*}\right)x_{1}^{*}x_{3}^{*}-n\theta_{2}g^{\prime }\left(x_{2}^{*}\right)x_{2}^{*}x_{3}^{*}\\ b_{5} & = & -\frac{nr_{1}\theta_{2}g^{\prime }\left(x_{2}^{*}\right)x_{1}^{*}x_{2}^{*}x_{3}^{*}}{k}-\frac{mr_{2}\theta_{1}f^{\prime }\left(x_{1}^{*}\right)x_{1}^{*}x_{2}^{*}x_{3}^{*}}{l}\\ & & +m\beta \theta_{2}g^{\prime }\left(x_{2}^{*}\right)x_{1}^{*}x_{2}^{*}x_{3}^{*}+n\alpha \theta_{1}f^{\prime }\left(x_{1}^{*}\right)x_{1}^{*}x_{2}^{*}x_{3}^{*}. \end{array}$$

In the presented work, the characteristic equation method will be used which, according to the work in [52], consists of the study of the stability of Equation (1) investigating the root distribution region of p(λ,τ)=0 in the complex plane. Note that the introduction of the delay parameter in the Equation (1) makes the stability analysis more complex. While in systems without delay, the number of roots of $p{\left(\lambda ,\tau \right)}_{{|}_{\tau =0}}$ is finite, for the case with τ≠0, the roots of p(λ,τ)=0, are infinite in number.

However, according to the work in [52], the roots of p(λ,τ)=0 with a positive real part, that is, Reλ(τ) such that p(λ(τ),τ)=0 are upper bound and isolated. Furthermore, the roots of the characteristic equation are isolated on the complex plane. We can then say that the quasi-polynomial p(λ,τ) given in Equation (9) has a finite number of roots with a positive real part. In particular, the roots of p(λ,τ) in any set of the form

$$\left\{\lambda =x+iy,\;a\leq x\leq b\right\},$$

with a,b∈R arbitrary real numbers are finite.

In what follows, we will use the *Pontryakin* theorem to obtain estimates of the network parameters that guarantee the asymptotic stability of Equation (1). For this we consider the following polynomials:

(11) $$\begin{array}{ccc} R(\omega ,\tau ) & = & Rep(i\omega ,\tau ),\\ S(\omega ,\tau ) & = & Imp(i\omega ,\tau ), \end{array}$$

what according to Equation (9) gives us

(12) $$\begin{array}{ccc} R(\omega ,\tau ) & = & -b_{1}\omega^{2}-b_{4}\omega \sin\left(\omega \tau \right)+b_{5}\cos\left(\omega \tau \right),\\ S(\omega ,\tau ) & = & -\omega^{3}+b_{2}\omega +b_{4}\omega \cos\left(\omega \tau \right)-b_{5}\sin\left(\omega \tau \right). \end{array}$$

According to the *Pontryakin* criterion the system, Equation (1), is asymptotically stable, if R(ω) and S(ω) have real, simple, intertwined roots, and the following condition is true for every real ω:

(13) $$T\left(\omega ,\tau \right)=R\left(\omega ,\tau \right)\frac{dS}{d\omega }\left(\omega ,\tau \right)-\frac{dR}{d\omega }\left(\omega ,\tau \right)S\left(\omega ,\tau \right)>0,$$

for more details on the concepts covered in the *Pontryakin* criterion see in [52]. From Equation (13) there are some highlighted points:

- the function T(ω,τ) establishes a relationship between the characteristic roots of the quasi-polynomial, Equation (9), and the system parameters, given by the coefficients $b_{i}$, i=1,…,5;
- from the inequality T(ω,τ)>0 it is possible to define regions of stability according to the equilibrium points, the system parameters and the delay time;
- the equilibrium points, and consequently the functional relationship T(ω,τ) depend on the sampling functions f,g;

Table 2 shows the equilibrium point convergence for the parameters, $m_{1}$, $n_{1}$, μ, $\theta_{i}$, $r_{i}$, *K*, *l*, α, β, *m*, *n*, i=1,2 and sample distribution functions f=sin(x),g=cos(x).

The numerical values of the reference parameters contained in the Table 2 were taken from [40]. The negative equilibrium values shows the system instability, meaning an amount of data above the processing capacity of the devices, in the case of WTIM’s and NCAP. Continuing with the analysis, the next step is determine the parameters $b_{i}$, i=1,…,5. This is done by replacing the parameters given in the Table 2 in Equation (10), to obtain the Table 3.

**Figure 13:**
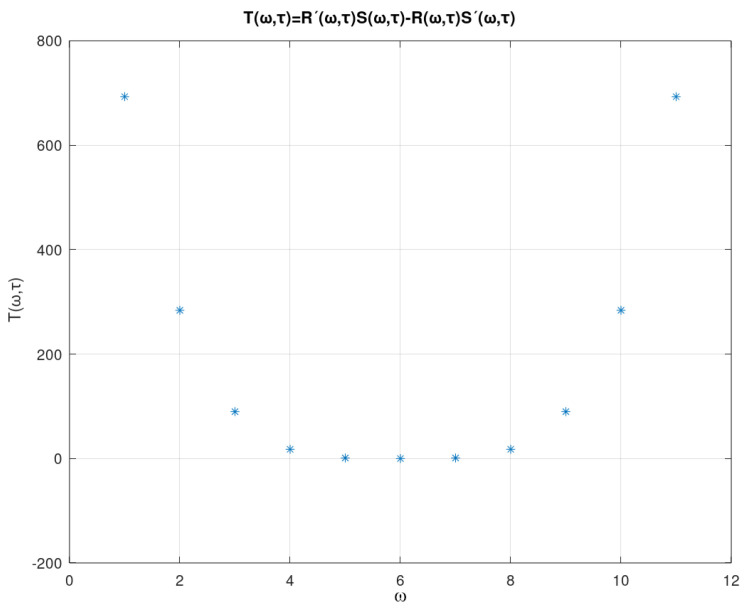
Quasi-characteristic polynomial.

**Figure 14:**
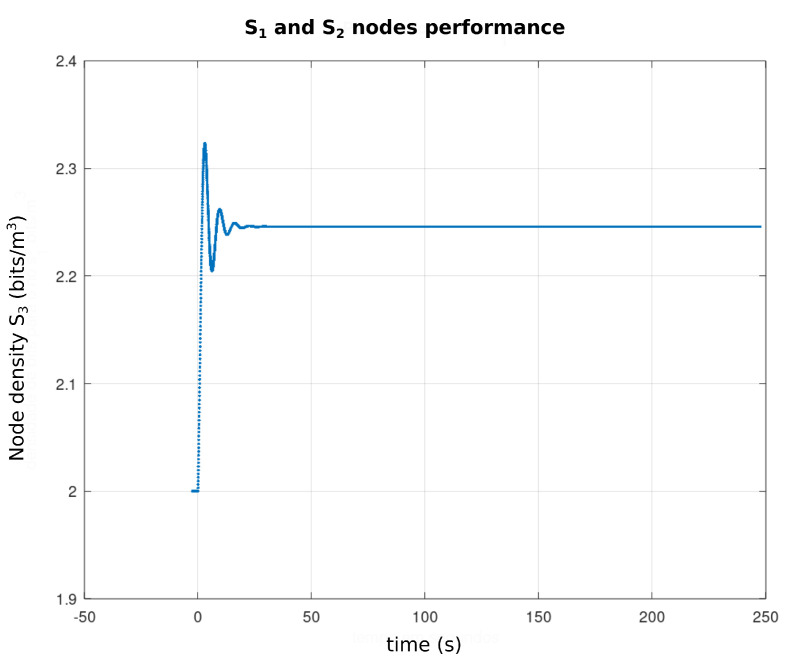
Performance graph of $S_{1}$ and $S_{2}$ nodes.

**Figure 15:**
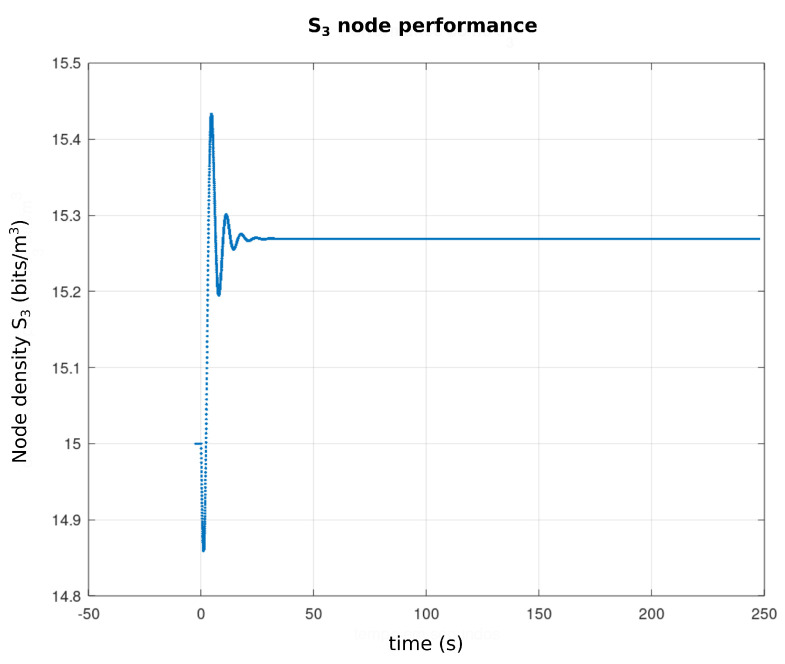
$S_{3}$ node performance graph.

**Figure 16:**
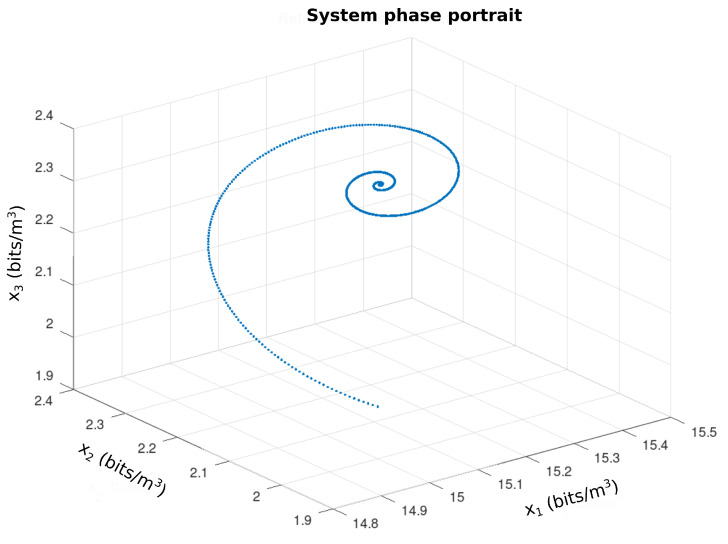
Phase picture of the sensor network.

Figure 13 represents the profile from T(ω,τ) to ω∈[−10,10] and τ∈[0,5] and the parameter vector $b_{i}$, i=1,…,5 given by the first line of the Table 3. As can be seen, T(ω,τ) has positive values which, according to the *Pontryagin* criterion, assure the stability of the equilibria. Based on this analysis, the Figure 14 shows the performance of nodes $s_{1}$ and $s_{2}$ (WTIM nodes), with respect to the number of bits per sample unit. The profiles presented show a stabilization of the number of bits per sample unit around 2.2 and 2.3 bits/sample unit for nodes $s_{1}$ and $s_{2}$, respectively, while Figure 15 shows a stabilization of the node $s_{3}$ (NCAP node) around 15.2 and 15.3 bits/sample unit. Figure 16 shows the performance of the three nodes with respect to the phase plane, in which presents a stable behavior converging to an equilibrium point, compatible with the values found, referring to the equilibrium points shown in the Table 2.

## 6. Conclusions

In this paper, we presented a mesh sensor network based on the IEEE 1451 standard, where the NCAP node in a plug-and-play manner detects the WTIM nodes, send requests to read the WTIM’s sensors, and routes the sensor data back in the network to the NCAP node that then stores the data. The nodes developed can communicate over long distances, without packet losses. Based on these characteristics, a mesh network can be easily expanded to perform a data acquisition in large areas without modifications in the network. The tests performed proved that the network is feasible for data collection purposes. In a future development, the plan is to insert a range of meteorological sensors in the WTIM’s, analyze power consumption, and to analyze the influence of meteorological conditions and external sign interference on communication. There are similar studies in the literature related to our proposal. However, in this paper the NCAP was implemented using a structured programming in a Raspberry pi B+ to be autonomous, extensible, in which the mesh network was evaluated in a real scenario, analyzing aspects of communication between the nodes. The work developed based on the IEEE 1451.1.2 and IEEE 1451.1.6 standard demonstrated the increased accessibility to the system from the external network side, being possible to be accessed through a web based system and IoT with a Bocker on the NCAP network node.

A dynamic model was also presented to describe the flow of communication between the NCAP and WTIMs. The numerical results confirm the existence of an equilibrium configuration with respect to the shared data flow between the NCAP and the WTIM.

## Figures and Tables

**Table 1:** Reading table of sensors and network data.

Date	Time	Temp. °C Low	RSSI dbm	Req/Resp Seconds	Press. Low	RSSI dbm	Req/Resp Seconds	Wind m/s	RSSI dbm	Req/Resp Seconds
14 November	00:30	19.29	−74	0.2313	915.591	−74	0.22888	0.2194	−74	0.23927
14 November	01:30	19.33	−74	0.27177	915.081	−74	0.21378	0.2277	−73	0.42216
14 November	02:30	19.52	−74	0.45759	915.086	−74	0.37362	0.5611	−75	0.23713
14 November	03:30	19.81	−75	0.49296	914.829	−74	0.47735	0	−74	0.08589
14 November	04:30	19.5	−74	0.2077	915.038	−74	0.2025	1.3111	−74	0.21133
14 November	05:30	19.44	−74	0.2277	915.346	−75	14 November	0.5777	−75	0.22465
14 November	06:30	19.59	−72	0.47874	915.639	−75	0.43566	0.4888	−74	0.49409
14 November	07:30	20.33	−74	0.43999	916.259	−75	0.47701	0.6305	−74	0.45841

**Table 2:** Variation of the balance points according to the network parameters.

$m_{1}$	$n_{1}$	μ	$\theta_{1}$	$\theta_{2}$	$r_{1}$	$r_{2}$	*K*	*l*	α	β	*m*	*n*	$x_{1}^{*}$	$x_{2}^{*}$	$x_{3}^{*}$
0.060	0.015	0.080	0.020	0.050	0.5	0.5	10	10	0.0080	0.0080	0.130	0.130	2.298	2.403	6.121
0.065	0.020	0.085	0.025	0.055	1.0	1.0	15	15	0.0085	0.0085	0.135	0.134	2.198	2.482	9.376
0.070	0.025	0.090	0.030	0.060	1.5	1.5	20	20	0.0090	0.0090	0.140	0.138	2.057	2.589	12.488
0.075	0.030	0.095	0.035	0.065	2.0	2.0	25	25	0.0095	0.0095	0.145	0.142	1.873	2.715	15.444
0.080	0.035	0.100	0.040	0.070	2.5	2.5	30	30	0.0100	0.0100	0.150	0.146	1.643	2.856	18.252
0.085	0.040	0.105	0.045	0.075	3.0	3.0	35	35	0.0105	0.0105	0.155	0.150	1.366	3.007	20.920
0.090	0.045	0.110	0.050	0.080	3.5	3.5	40	40	0.0110	0.0110	0.160	0.154	1.043	3.165	23.459
0.095	0.050	0.115	0.055	0.085	4.0	4.0	45	45	0.0115	0.0115	0.165	0.158	0.673	3.328	25.878
0.100	0.055	0.120	0.060	0.090	4.5	4.5	50	50	0.0120	0.0120	0.170	0.162	0.258	3.494	28.187

**Table 3:** Value of $b_{i}$ parameters.

$b_{1}$	$b_{2}$	$b_{3}$	$b_{4}$	$b_{5}$
0.313	0.187	0.022	0.214	0.009
0.351	0.312	0.044	0.394	0.015
0.371	0.450	0.071	0.646	0.023
0.382	0.596	0.098	0.998	0.029
0.385	0.742	0.123	1.481	0.034
0.382	0.878	0.140	2.130	0.037
0.374	0.995	0.141	2.983	0.039
0.360	1.081	0.117	4.074	0.038
0.341	1.122	0.056	5.406	0.025

## Data Availability

Not applicable.

## Abbreviations

The following abbreviations are used in this manuscript:

API	Application Programming Interface
CIoT	Critical IoT
CL	Criticality Level
FPGA	Field Programmable Gate Array
GPIO	General Purpose Input/Output
GPU	Graphics Processing Unit
HART	Highway Addressable Remote Transducer
HDMI	High-Definition Multimedia Interface
HTTP	Hypertext Transfer Protocol
I2C	Inter-Integrated Circuit
IEEE	Institute of Electrical and Electronic Engineers
IoT	Internet of Things
LED	Light Emitting Diode
MAC	Media Access Control
MCC	Mobile Cloud Computing
MQTT	Message Queuing Telemetry Transport
MQTT-SN	Message Queuing Telemetry Transport-Sensor Network
NCAP	Network Capable Application Processor
PHP	Hypertext Preprocessor
RAM	Random Access Memory
RDF	Resource Description Framework
RED	Random Early Detection
RFID	Radio-Frequency Identification
RSSI	Received Signal Strength Indication
SL	Security Level
SMNP	Simple Network Management Protocol
SPARQL	SPARQL Protocol and RDF Query Language
SPI	Serial Peripheral Interface
SSW	Semantic Sensor Web
TCP/UDP	Transmission Control Protocol/User Datagram Protocol
TEDS	Transducers Electronic Data Sheet
UART	Universal Asynchronous Receiver/Transmitter
USB	Universal Serial Bus
UTF8	8-bit Unicode Transformation Format
VHDL	VHSIC Hardware Description Language
VHSIC	Very-High-Speed Integrated Circuit
WMN	Wireless Mesh Network
WSN	Wireless Sensor Network
WTIM	Wireless Transducer Interface Module
XML	Extensible Markup Language
XMPP	Extensible Messaging and Presence Protocol
